# Comparative assessment and novel strategy on methods for imputing proteomics data

**DOI:** 10.1038/s41598-022-04938-0

**Published:** 2022-01-20

**Authors:** Minjie Shen, Yi-Tan Chang, Chiung-Ting Wu, Sarah J. Parker, Georgia Saylor, Yizhi Wang, Guoqiang Yu, Jennifer E. Van Eyk, Robert Clarke, David M. Herrington, Yue Wang

**Affiliations:** 1 Department of Electrical and Computer Engineering, Virginia Polytechnic Institute and State University, 900 N. Glebe Road, Arlington, VA 22203 USA; 2 Cedars Sinai Medical Center, Advanced Clinical Biosystems Research Institute, Los Angeles, CA 90048 USA; 3 Department of Internal Medicine, Wake Forest University, Winston-Salem, NC 27157 USA; 4 The Hormel Institute, University of Minnesota, Austin, MN 55912 USA

**Keywords:** Computational biology and bioinformatics, Proteome informatics

## Abstract

Missing values are a major issue in quantitative proteomics analysis. While many methods have been developed for imputing missing values in high-throughput proteomics data, a comparative assessment of imputation accuracy remains inconclusive, mainly because mechanisms contributing to true missing values are complex and existing evaluation methodologies are imperfect. Moreover, few studies have provided an outlook of future methodological development. We first re-evaluate the performance of eight representative methods targeting three typical missing mechanisms. These methods are compared on both simulated and masked missing values embedded within real proteomics datasets, and performance is evaluated using three quantitative measures. We then introduce fused regularization matrix factorization, a low-rank global matrix factorization framework, capable of integrating local similarity derived from additional data types. We also explore a biologically-inspired latent variable modeling strategy—convex analysis of mixtures—for missing value imputation and present preliminary experimental results. While some winners emerged from our comparative assessment, the evaluation is intrinsically imperfect because performance is evaluated indirectly on artificial missing or masked values not authentic missing values. Nevertheless, we show that our fused regularization matrix factorization provides a novel incorporation of external and local information, and the exploratory implementation of convex analysis of mixtures presents a biologically plausible new approach.

## Introduction

Liquid chromatography coupled to mass spectrometry (LC–MS) is a popular method for high-throughput identification and quantification of thousands of proteins in a single analysis^1,2^. The LC–MS signals can be displayed in a three-dimensional space consisting of the mass-to-charge ratios, retention times and intensities for the observed peptides. However, this approach suffers from many missing values at the peptide or protein level, which significantly reduces the amount of quantifiable proteins with an average of 44% missing values from traditional LC–MS workflows^3–5^.

While there are multiple causes for this missingness, three typical missing mechanisms are widely acknowledged. Low abundant proteins may be missing because their concentration is below the lower limit of detection (LLOD); while poorly ionizing peptides or problems in technical pre-processing may cause proteins to be missing not at random (MNAR)^6^. However, missingness may also extend to mid- and even high-range intensities^5^, statistically categorized into missing at random (MAR) and missing completely at random (MCAR)^7^. MAR is actually missing conditionally at random given the observed data distribution or underlying parametric covariates. MCAR depends on neither observed nor missing data, thus the incomplete data are representative of the entire dataset. While MAR allows prediction of the missing values based on observed data, unfortunately, the MAR and MNAR conditions cannot be distinguished based on the observed data because by definition missing values are unknown^8,9^. More importantly, missing values in reality can originate from a mix of both known and unknown missing mechanisms^7,10^.

A common solution for missingness is to impute the missing values based on assumed missing mechanisms. However, this approach can introduce a profound change in the distribution of protein-level intensities because most methods are only designed for a single missing mechanism. These changes can have unpredictable effects on downstream differential analyses. While many imputation methods have been adopted for imputing missing values in proteomics data, comparative evaluation of their relative performance remains inconclusive. Moreover, few studies provide an outlook on how best to address unresolved problems or future development directions^4,9,10^.

To better understand the strengths and limitations of both imputation methods and assessment designs, we conduct a collective assessment of eight representative methods involving three typical missing value mechanisms in conjunction with authentic missing values. Compared using a set of realistic (preserving data distribution) simulations derived from real proteomics data sets, the performance of the selected methods is measured by three criteria^11^, root-mean-square error (RMSE), normalized root-mean-square error (NRMSE), and sum of ranks (SOR). Several important observations are evident from this comparison study. First, while imputation methods perform differentially under various missing mechanisms, algorithmic parameter settings, and preprocessing procedures, some methods consistently perform better than others across a range of realistic simulation studies. Second, the quality of performance assessment depends on the efficacy of simulation designs; a more realistic simulation design should include authentic missing values and preserve the original overall data distribution. Third, existing assessment methodologies are imperfect in that performance is indirectly assessed on imputing either artificial or masked, but not authentic missing values (see Discussion section).

To explore a more integrative strategy for improving imputation performance, we discuss a low-rank matrix factorization framework with fused regularization on both sparsity and similarity—Fused Regularization Matrix factorization (FRMF)^12–14^, which can naturally integrate other-omics data such as gene expression or clinical variables. We also introduce a biologically-inspired latent variable modeling strategy—Convex Analysis of Mixtures (CAM)^14,15^, which explicitly formulates a data matrix as mixtures of underlying biological archetypes and performs missing value imputation on original intensity data (before log-transformation). Preliminary results on real proteomics data are provided together with an outlook into future development directions.

## Results

### Experimental design and protocol

**Figure 1:**
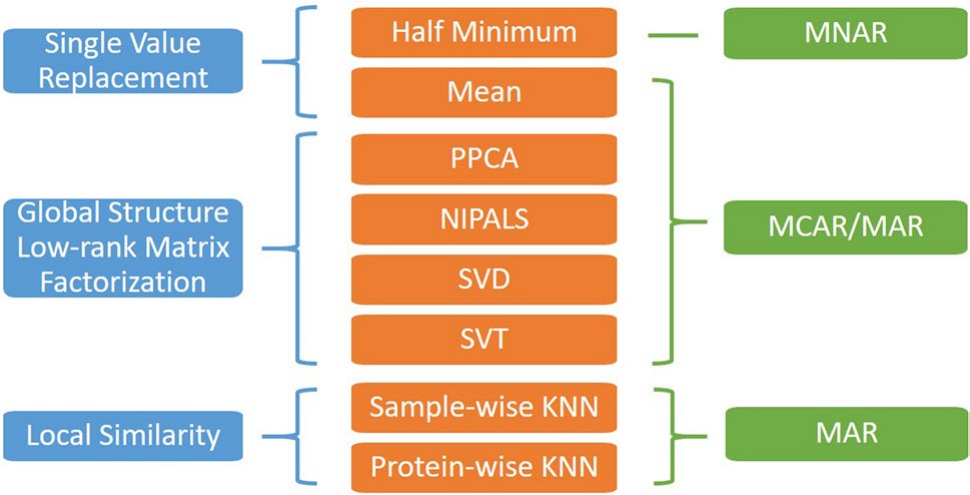
Comparative assessment of eight representative missing value imputation methods, divided into three categories.

We selected eight representative methods for comparative assessment, based on their intended missing mechanism(s) and imputation principles (summarized in Fig. 1). One method (Min/2) is devoted to MNAR (LLOD)^7^, two methods (swKNN and pwKNN) are tailored to MAR (local-similarity)^16^, and five methods (Mean, PPCA, NIPALS, SVD, and SVT) are intended for MCAR/MAR (global-structure or low-rank matrix factorization)^7,10,17–19^. We then explored and tested several variants of FRMF and CAM, where local similarity information is obtained from baseline or other data acquired from the same samples.

**Figure 2:**
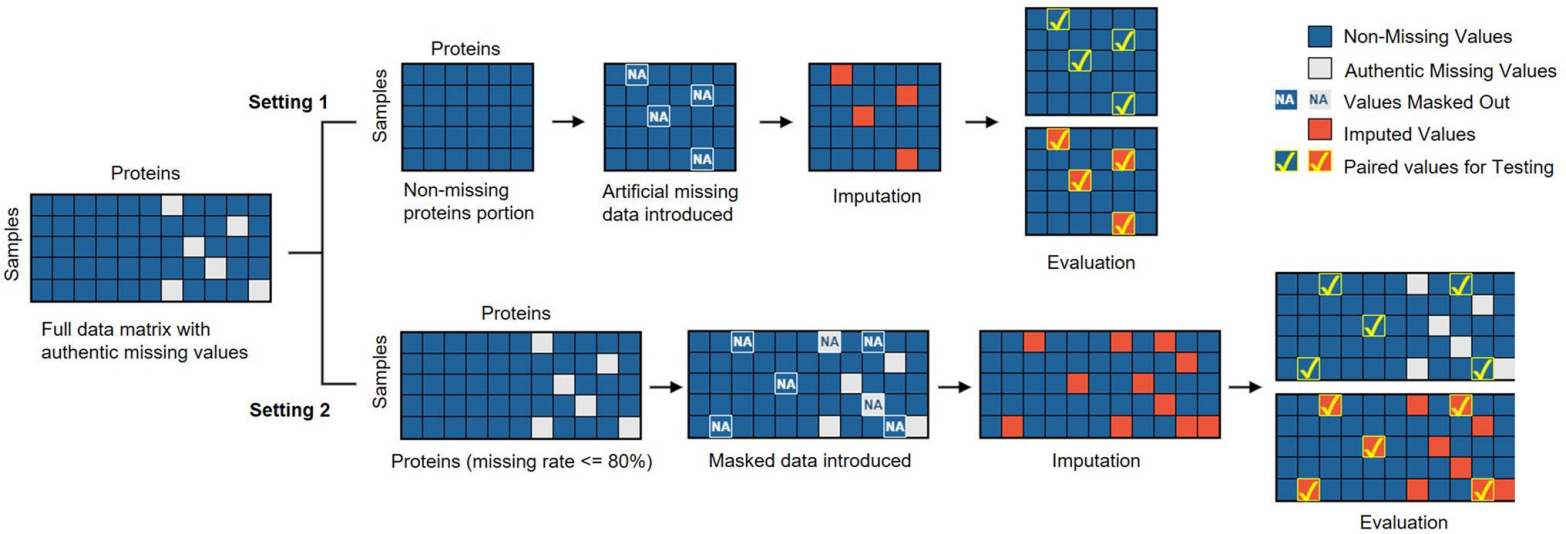
Two-phased workflow of realistic simulation-based assessment on missing value imputation methods.

We conducted the comparative assessments in two complementary simulation settings. In simulation setting 1, the simulation data were generated from the observed data portion (no authentic missing value) of a real proteomics dataset, where artificial missing values were introduced by two typical missing mechanisms and used for performance assessment. In simulation setting 2, the simulation data were generated from the complete data matrix (including authentic missing values) of a real proteomics dataset, where a small percentage of data points were randomly set-aside (masked values) and used solely for performance assessment. Preprocessing eliminates proteins with missing rates higher than 80% and then performs log2 transformation^5^. Parameters were optimized for each imputation method by parameter sweeping over a wide range of settings at each missing rate. The overall experimental workflow is given in Fig. 2.

### Real proteomics data

Real LC–MS proteomics data form the base from which the simulation data sets were produced^6^. Data were acquired using label-free data-independent acquisition (DIA) protocol, and protein level output was generated by mapDIA^20^. The resulting dataset contains 200 samples associated with 2682 proteins measured in human left anterior descending (LAD) coronary arteries collected as part of a study of coronary and aortic atherosclerosis^21^. Data were produced in three separate batches, indexed as A, B, and C; and all data passed the quality control and preprocessing procedures^6,21^, as summarized in Table 1 (Supplementary Information). To avoid unknown and unnecessary batch effects, all simulation datasets were generated from batch A dataset that has the largest sample size (n = 98). For the simulations on other batches, please see the Supplementary Information.

**Table 1:** Summary of real proteomics datasets used in this work (DIA-MS).

	Sample size	Protein size	Total Missing Rate #MV/(#Sample*#Protein)	Setting #1 protein size (non-missing proteins)	Setting #2 protein size (proteins with ≤ 80% missing rate)
Batch A	98	2107	24.67%	751 (35.64%)	1935 (91.84%)
Batch B	55	2604	29.63%	819 (31.45%)	2324 (89.25%)
Batch C	47	2590	25.52%	976 (37.68%)	2325 (89.77%)

### Simulation data generated from the observed portion of the data matrix (Setting 1)

Based on the observed portion of data matrix (without authentic missing values), we adopted a hybrid missing data model and used the R package imputeLCMD to introduce artificial missing values while preserving the original observed data patterns^22^. Specifically, MCAR missing values were introduced by randomly replacing some data points with ‘NA’ (not available) according to the designed missing rates (approximately from 1 to 50%); MNAR missing values were introduced by quantile cut-off for the full dataset^7,10,23^; and mixed MCAR and MNAR missing values were introduced by assigning $(1-\beta )$ portion of MCAR and $\beta$ portion of MNAR; corresponding to missing rate $\alpha$ and $\beta =0, 0.1, 1$ (Supplementary Information).

### Simulation data with set-aside masked values from the full data matrix (Setting 2)

In this simulation setting, we used the full data matrix (including both observed and authentic missing values) from the human coronary proteomics dataset. To preserve the original patterns of both observed and authentic missing values, for each protein, a small percentage of data points in the complete data matrix were randomly set-aside as ‘NA’ (masked values) with the masking rate(s) proportional to the authentic missing rate(s). This procedure was repeated for all proteins and the masked values were considered as a mix of MNAR and MAR conditioned on the observed missing rates and data patterns. The resulting total missing rates (authentic and masked) versus mean expressions are shown in Figure S5, preserving well the overall patterns of the original full data matrix (Supplementary Information).

### Performance assessment focused on MNAR (Setting 1)

**Figure 3:**
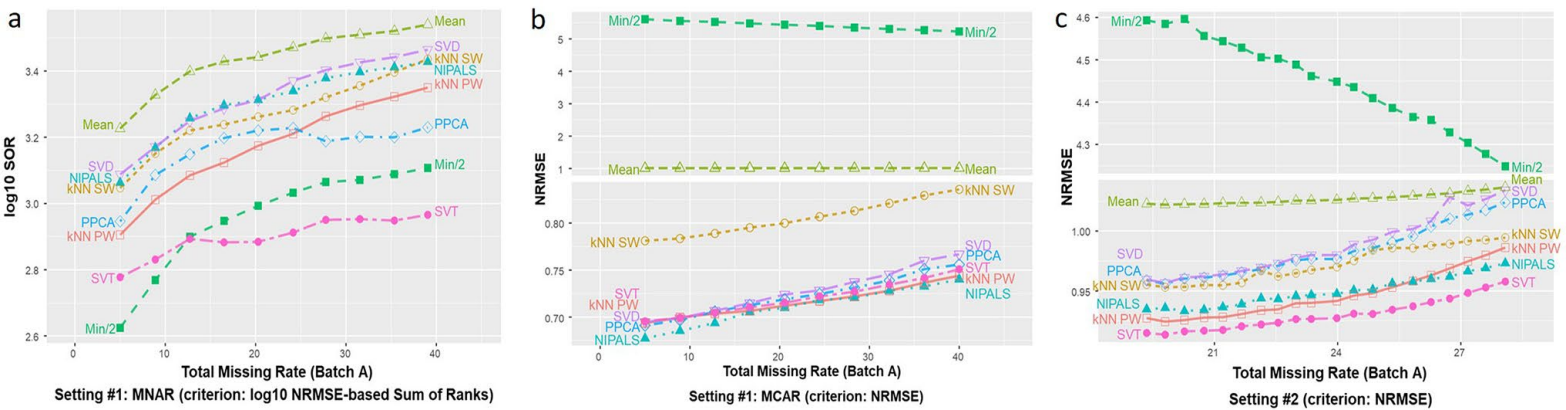
(**a**) Imputation performance of the eight methods on the simulation data of setting #1, with assumed MNAR missing mechanism and varying total missing rates. (**b**) Imputation performance of the eight methods on the simulation data of setting #1, with assumed MCAR missing mechanism and varying total missing rates. (**c**) Imputation performance of the eight methods on the simulation data of setting #2, focusing on authentic missing mechanism and varying masked rates.

As shown in Fig. 3a, under an MNAR missing mechanism assumption, SVT and Min/2 yielded the best performance, and the relative performance of SVT and Min/2 depends on the missing rates and criterion used for evaluation. It is important to reiterate that in reality the MAR and MNAR conditions cannot be distinguished based on the observed data because by definition missing values are unknown^8,9^. As expected, the MNAR-devoted method, Min/2, performs much better than the others. The baseline method Mean performs worst among all methods (see additional results in Supplementary Information). Note that SOR increases expectedly when the missing rate increases because SOR is positively associated with the number of missing proteins, therefore the value of SOR may not imply the absolute performance of a method. Additional experimental results are given in Figure S2 and Figure S6a (multiple trials).

### Performance assessment focused on MCAR (Setting 1)

Imputation performance of the eight methods on the MCAR mechanism is summarized in Fig. 3b. Experimental results show that NIPALS performs better than all other methods and for almost all three evaluation criteria. SVT is the best method when RMSE is used. Min/2 performs the worst among all other methods in all cases, likely due to its design for the MNAR mechanism. Mean performs consistently poorly over different missing rates, with only except for Min/2 showing a worse performance. While all methods perform worse when the total missing rate increases, the ranking of their relative performances remains unchanged (see additional results in Supplementary Information). Additional experimental results are given in Figure S1, Figure S3, and Figure S6b (multiple trials).

### Performance assessment focused on authentic missing values (Setting 2)

As shown in Fig. 3c, NIPALS, SVT, and protein-wise or sample-wise KNN achieve the best performance, where authentic missing values are dominant and where imputation accuracy is evaluated on the masked values. This observation is consistent with what has been reported previously^5,6,11^. As expected, the MNAR-devoted method Min/2 has the worst performance. Similar to the case of MCAR, low-rank methods and local-similarity methods perform worse when the total missing rate increases, and among these methods, SVD and PPCA perform worse than the baseline method Mean when the total missing rate is large. Note that because low abundant proteins often have higher authentic missing rates and accordingly higher masking rates, more low abundant proteins (possibly the minimum values) are masked than highly expressed proteins. Thus, the counterintuitive decrease in NRMSE by Min/2 is expected when the authentic missing rate increases. Additional experimental results are given in Figure S4 and Figure S6c (multiple trials).

The relative imputation performance among these representative existing methods is summarized in Table 2, assessed by three performance measures and over three data batches.

**Table 2:** Summary of the relative performance among the imputation methods being evaluated.

Overall summary (Batch A, Batch B, Batch C)						
Mechanisms	Best			Worst		
	Criteria					
	NRMSE	RMSE	SOR	NRMSE	RMSE	SOR
Setting #1						
MCAR	NIPALS SVT KNN PW	SVT NIPALS KNN PW	NIPALS	HalfMin	HalfMin	HalfMin
MIX (90% MCAR + 10%MNAR)	SVT NIPALS KNN PW	SVT NIPALS KNN PW	SVT NIPALS KNN PW	HalfMin	HalfMin	HalfMin
MNAR	SVT HalfMin	HalfMin	SVT HalfMin	Mean	Mean	Mean
Setting #2						
MAR + MNAR	SVT KNN SW NIPALS	SVT	NIPALS SVT KNN SW	HalfMin	HalfMin	HalfMin

### Evaluation of the FRMF method focused on authentic missing values (Setting 2)

In this study we aimed to experimentally test whether the FRMF method that integrates local similarity derived from within and/or external data could improve imputation accuracy as compared with global low-rank SVD, an existing approach based on a similar principle. We evaluated three variants of the FRMF method. RMF serves as a baseline sparsity regularized matrix factorization algorithm^13^; FRMF_self introduces a fused-regularization utilizing the similarity among samples embedded within the data matrix; and FRMF_cross_patho exploits external pathological scores using a fused-regularization strategy where the pathological scores are the qualitative percentages of the intimal surface involvement of various atherosclerotic changes graded by pathologists^6^.

**Figure 4:**
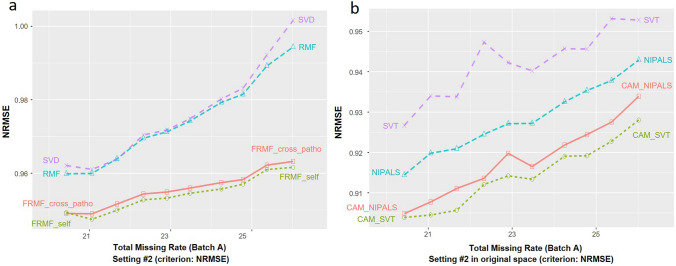
(**a**) Imputation performance of the FRMF variants on the simulation data of setting #2, with varying masked rates. (**b**) Imputation performance of CAM variants on the simulation data of setting #2, with varying masked rates, in comparison to that of SVT and NIPALS. The imputation accuracy is evaluated in the original intensity space (before log-transformation).

The experimental results are shown in Fig. 4a. While RMF performs comparably with SVD and starts to perform better when missing rates are larger than 25%, both FRMF_self and FRMF_cross_patho performs significantly better than RMF. This preliminary result implies a potential benefit for combining global low-rank and local-similarly regularizations, and also for leveraging external information via fused regularization. Additional experimental results on multiple trials are given in Figure S7a.

### Evaluation of the CAM method focused on authentic missing values (Setting 2)

In this study we aimed to experimentally test whether the CAM method that exploits biologically interpretable latent variable models in the matrix factorization could improve imputation accuracy as compared with the top existing approaches that use a similar principle (i.e. SVT; NIPALS). Accordingly, based on biologically-inspired latent variable modeling of complex tissues^14,15^, we proposed and evaluated three variants of a CAM based imputation strategy. CAM_complete performs CAM based imputation using the non-missing portion of full data matrix; CAM_SVT and CAM_NIPALS perform CAM based imputation using full data matrix where the input data matrix is initialized by either SVT or NIPALS, respectively.

The experimental results are shown in Fig. 4b and Figure S9a. As expected, CAM_complete performs much better than the baseline method Mean. More importantly, both CAM_NIPALS and CAM_SVT consistently performs better than NIPALS and SVT—the two top performers from our earlier comparative assessment. This preliminary result shows that biologically-plausible latent variable modeling may potentially improve imputation accuracy within the framework of low-rank optimization. Additional experimental results on multiple trials are given in Figure S7b.

## Discussion

The ability to simulate the missing mechanisms (MNAR, MAR, or MCAR) depends on the efficacy of the tools applied. However, because simulation relies on the statistics estimated from the observed portion of a data matrix, and so the artificial missing values introduced cannot fully resemble authentic missing mechanisms and/or patterns present in the original overall data distribution. More critically, performance can only be assessed on evaluating the imputed artificial not authentic missing values, because authentic missing values are intrinsically unknown and the overall data distribution may be distorted by the introduced artificial missing values. While it may be informative to compare the impact of the imputation versus non-imputation on some subsequent data analysis in the future, we have opted to focus on assessing direct imputation accuracy, because the evaluation using subsequent analysis would be indirect and task-dependent.

To address the aforementioned issues in the presence of authentic missing values, a small percentage of set-aside values were introduced into the complete data matrix and used solely for the purpose of assessment. Because masked values are randomly assigned onto both observed and authentic missing values, the simulation maximally preserves the original overall data distribution. Masked values may represent a mix of MNAR (high missing rate associated with low protein abundance) and MAR (joint distribution of both observed and authentic missing values). However, performance is assessed indirectly on imputing masked not authentic missing values. An interesting addition may be to include the performance accuracy in estimating non-missing values by the imputation function.

Imputation accuracy could be affected by data preprocessing and algorithmic parameter setting. In this study, sample-wise normalization and protein-wise standardization are performed based on the requirements of each method. Data preprocessing affects the scale of NRMSE, but relative performance across various methods remains consistent. While imputation performance varies with parameter setting, there is no theoretical guideline for optimizing the parameter setting. Nevertheless, a cross-validation approach could be used to optimize algorithms parameters in future work. Here, we used grid search to tune the parameters based on the consideration that when all possible parameters are enumerated the optimal setting can be reached. Moreover, other correlation-based relative performance measures (Pearson and/or Spearman) can be calculated on introduced missing or masked values and non-missing values.

The observation that imputation methods based on low-rank matrix factorization (e.g. SVT or NIPALS) perform consistently better than other ones (in both simulation settings) is consistent with the reports from other similar evaluation studies^4,11^. While the true missing mechanisms in proteomics data are unknown and hard to simulate, the better performance of SVT or NAPALS may be expected particular in the cases with relatively small sample size. Missing value imputation is principally an unsupervised learning task, and the sample size is determined by the number of observed values over both samples and proteins. Concerning unavoidable noise/outlier and sample heterogeneity embedded within the observed values of data matrix, related to possibly much smaller ‘effective’ sample size, SVT or NIPALS leverages low-rank regularization to avoid potential overfit of the model to noise/outlier.

FRMF is a novel integrated imputation approach with promising preliminary results. However, the effectiveness of FRMF for improving global low-rank matrix completion methods depends on both sample diversity and the complementary nature of additional and relevant measurements such as the complementary roles of local similarity and global structure. For example, FRMF imputation may be performed on combined biologically diverse sample groups, or local similarity derived from gene expressions may be incorporated to impute protein missing values on the same samples.

The CAM method we propose represents a new direction for future work. For example, CAM may be integrated into FRMF to leverage local similarity derived from complementary information on the same samples and the input data matrix for CAM may be initialized by missing-value-insensitive NMF (nonnegative matrix factorization)^12^. More importantly, CAM performs missing value imputation using the original intensities rather than log-transformed data. This approach is both biologically plausible and mathematically more rigorous because log-transformation violates the linear nature of low-rank matrix factorization^24^.

There are two major approaches for generating bottom-up MS proteomic data: DIA and DDA (data-dependent acquisition). Therefore, we also applied FRMF and CAM methods to the simulation datasets (setting #2) based on a DDA dataset produced by the Orbitrap Elite analyzer coupled to an Easy-nLC 1000 system (Thermo Scientific), followed by tandem mass spectra of the 20 most abundant peaks in the linear ion trap. This DDA dataset is associated with the same study samples (Table S1). The experimental results are given in Figure S8 and Figure S9b. It can be seen that FRMF variants consistently outperform peer methods including SVD and RMF, and CAM_NIPALS performs slightly better than SVT while worse than NIPALS. We would like to reiterate that while MAR allows prediction of missing values based on observed data, unfortunately, the MAR and MNAR conditions cannot be distinguished based on the observed data because by definition missing values are unknown. Given that DDA data type has higher missing rates (Table S1), expectedly, the CAM method may not perform well because biologically interpretable basis may not be correctly estimated due to missing marker genes and critical gene patterns that cannot be recovered by SVT.

In future work, support vector machine or artificial neural network (ANN) based methods may be considered as emerging imputation competitors^11^, and a combination approach utilizing an ensemble of strategies could be explored^4^. We have recently begun to explore a deep matrix completion method^25^. Furthermore, some advanced hyperparameter auto-search tools could be adapted to optimize the hyperparameters often embedded within various imputation algorithms^26^. Conclusions and insights from more recent similar evaluation work should also be considered^11^.

## Method

### Brief introduction to the eight existing methods

- **Min/2 (half minimum)** Taking MNAR as the missing value mechanism, for each protein the missing values are estimated as half the minimum value of the observed intensities in that protein across all samples^6,9^.
- **Mean** For MAR/MCAR as the missing value mechanism, for each protein we replaced the missing values with the mean value of the observed intensities in that protein across all samples^6,9^.
- **swKNN (sample-wise k-nearest neighbors)** Taking MAR as the missing value mechanism, we leveraged local similarity among samples for each protein, replacing the missing values with the weighted average of observed intensities in that protein proportional to the proximities of k-nearest neighboring samples^9^.
- **pwKNN (protein-wise k-nearest neighbors)** Where MAR was the presumed missing value mechanism, we leveraged local similarity among proteins for each sample, replacing the missing values with the weighted average of observed intensities in that sample proportional to the proximities of k-nearest neighboring proteins (with protein-wise normalization)^9^.
- **PPCA (probabilistic PCA)** For MCAR/MAR as the missing value mechanism, a low-rank probabilistic PCA matrix factorization was estimated by the expectation maximization (EM) algorithm and then used to impute missing values^27^.
- **NIPALS (non-linear estimation by iterative partial least squares)** Taking MCAR/MAR as the missing value mechanism, a low-rank missing-data-tolerant PCA matrix factorization was estimated by iterative regression and then used to impute missing values^28,29^.
- **SVD (SVDImpute)** For MCAR/MAR as then missing value mechanism, a low-rank SVD matrix factorization was estimated by the EM algorithm and used to impute missing values^28,30^.
- **SVT (singular value thresholding)** Where we assumed MCAR/MAR to be the missing value mechanism, a low-rank SVT matrix factorization was estimated by iteratively solving a nuclear norm minimization problem and then used to impute missing values^19^.

### Background information of the data utilized

Coronary artery disease remains a leading cause of death in industrialized nations, and early detection of disease is a critical intervention target to effectively treat patients and manage risk. The early detection of atherosclerotic status and burden in unselected populations by circulating factors remains limited, with a pressing need for sensitive and specific circulating biomarkers to more accurately estimate risk of major adverse cardiac events and target high risk individuals for early preventive interventions. We quantified the proteome of 99 paired abdominal aorta (AA) and left anterior descending coronary artery (LAD) specimens (N = 198 specimens total) acquired during autopsy of young adults free of diagnosed cardiac disease. Trained pathologist scored each specimen for surface involvement of fatty streak or fibrous plaque/calcified lesions^6^.

Specimens were flash frozen in liquid nitrogen and stored until further processing. Each tissue was pulverized in liquid nitrogen and homogenized. Protein concentration of the supernatant was assessed by CB-X assay kit (G-Biosciences MO, USA), and 15 μg of protein was aliquoted and precipitated using 2-D cleanup kit (GE Healthcare MA, USA) and then reconstitute in 6 M urea, 50 mM ammonium bicarbonate. We used both data-dependent acquisition (DDA) and DIA acquisitions to maximize peptide detection and protein identification. To generate a peptide spectral library for subsequent identification and quantification of peptides and proteins, peptides from representative specimen were pooled and separated into 80 basic reverse phase fractions. These were analyzed by DDA mass spectrometry analysis for the assembly of a human vascular peptide assay library. Peptide peak groups were extracted from an existing library of pooled human vascular lysates described previously and can be accessed at http://www.peptideatlas.org/PASS/PASS01066, using the openSWATH workflow^21^. In the discovery phase, proteome of these specimens with varying degrees of lesion was analyzed as quantified by label-free spectral counting of DDA-MS data^6^. To facilitate rapid transition from discovery into translation, we employed DIA-MS, for which a targeted peptide peak group readily exists for each peptide identified from a protein of interest detected in discovery phase^21^.

### Performance measures

Three quantitative measures were used to evaluate imputation accuracy, namely Root Mean Square Error (RMSE), Normalized Root Mean Square Error (NRMSE), and Sum of Ranks (SOR). Specifically, RMSE and NRMSE are given by^31,32^

$${\text{RMSE}} = { }\sqrt {\frac{{\mathop \sum \nolimits_{{\Omega }} \left( {\hat{\varvec{X}}_{{\Omega }} - \varvec{X}_{{\Omega }} } \right)^{2} }}{{\left| {\Omega } \right|}}} ,\quad {\text{NRMSE}} = { }\sqrt {\frac{{\mathop \sum \nolimits_{{\Omega }} \left( {\hat{\varvec{X}}_{{\Omega }} - \varvec{X}_{{\Omega }} } \right)^{2} }}{{\left| {\Omega } \right|\sigma_{{\varvec{X}_{{\Omega }} }}^{2} }}} ,$$

respectively, where $\Omega$ is the index set of missing values in complete data matrix $\varvec{X}$, $|\Omega |$ is the total number of missing values, $\hat{\varvec{X}}$ is the imputed complete data matrix, and ${\sigma }_{\varvec{X}_{\Omega }}^{2}$ is the variance of missing values. To address the bias of NRMSE with the MNAR missing mechanism, SOR has been proposed as^23^.

$${\text{SOR}} = \sum\nolimits_{(i = 1)}^{P} {rank({\text{NRMSE}}_{{\text{i}}} )} ,$$

where $P$ is the number of proteins containing at least one missing value, $i$ is the protein index in this protein subset, and $rank({\mathrm{NRMSE}}_{i})$ is the ranks of protein-wise NRMSE across different imputation methods.

### Introduction to FRMF method

Low-rank matrix factorization is a popular and effective approach for missing data imputation^13^. For imputing proteomics data, the assumption is that there is only a small number of biological processes determining the expression profiles. This fundamental assumption is biologically plausible because measured abundances in a given sample contain substantial information of other unobserved proteins, and information of other samples with shared properties can also be useful in the learning step^4,11^. Consider an $m\times n$ complete data matrix ${\varvec{X}}$ describing $m$ samples and $n$ proteins. A low-rank matrix factorization approach seeks to approximate ${\varvec{X}}$ containing missing values by a linear latent variable model,

1 $${{\varvec{X}}}_{m\times n}= {{\varvec{A}}}_{m\times l}{\times {\varvec{S}}}_{l\times n},$$

where ${{\varvec{A}}}_{m\times l}$ and ${{\varvec{S}}}_{l\times n}$ are the low-rank factor matrices, and $l\ll \mathrm{min}(m,n)$. To prevent overfitting, the solution is often formulated as a regularized sparse SVD minimization problem on the observed values

2 $$\mathrm{min}\sum_{i=1}^{m}\sum_{j=1}^{n}I\left(\varvec{X}_{ij}\ne \mathrm{NA}\right){\left(\varvec{X}_{ij}-{{\varvec{A}}}_{i}{{\varvec{S}}}_{j}\right)}^{2}+{\lambda }_{\mathrm{A}}{\Vert {\varvec{A}}\Vert }_{F}^{2}+{\lambda }_{\mathrm{S}}{\Vert {\varvec{S}}\Vert }_{F}^{2},$$

where ${\Vert .\Vert }_{F}^{2}$ denotes the Frobenius norm, $I\left(.\right)$ is the indicator function, and ${\lambda }_{\mathrm{A}},{\lambda }_{\mathrm{S}}>0$ are the regularization parameters. When local similarity information is available^25^, FRMF can be reformulated by adding a fused regularization term

3 $$\mathrm{min}\sum_{i=1}^{m}\sum_{j=1}^{n}I\left(\varvec{X}_{ij}\ne \mathrm{NA}\right){\left(\varvec{X}_{ij}-{{\varvec{A}}}_{i}{{\varvec{S}}}_{j}\right)}^{2}+{\lambda }_{\mathrm{A}}{\Vert {\varvec{A}}\Vert }_{F}^{2}+{\lambda }_{\mathrm{S}}{\Vert {\varvec{S}}\Vert }_{F}^{2}+\alpha \sum_{i=1}^{m}\sum_{k\in \mathcal{F}(i)}{\Vert \varvec{A}_{i}-\varvec{A}_{k}\Vert }_{F}^{2},$$

where $\alpha$ is the fused regularization parameter, and $\mathcal{F}(i)$ denotes the neighborhood sample subset of sample $i$ and can be determined using baseline data or other relevant measurements such as gene expression or pathological score. In our study, $\mathcal{F}(i)$ was determined by the between-sample cosine similarity $\mathrm{cos}(\varvec{X}_{i},\varvec{X}_{k})$ based on data matrix in FRMF_self, or $\mathrm{cos}(\varvec{P}_{i},\varvec{P}_{k})$ based on external pathological scores on samples in FRMF_cross_patho.

Because pair-wise local similarity among samples has already been exploited for determining neighborhood $\mathcal{F}(i)$, we adopted the average-based fused regularization^13^. A local minimum of the objective function given by Eq. 3 can be found by performing gradient descent in latent variable vectors ${{\varvec{A}}}_{i}$ and ${{\varvec{S}}}_{j}$,

4 $$\frac{\partial Err}{\partial {{\varvec{A}}}_{i}}=\sum_{j=1}^{n}I\left(\varvec{X}_{ij}\ne \mathrm{NA}\right)\left(\varvec{X}_{ij}-{{\varvec{A}}}_{i}{{\varvec{S}}}_{j}\right){{\varvec{S}}}_{j}+{\lambda }_{\mathrm{A}}{{\varvec{A}}}_{i}+\alpha \sum_{k\in \mathcal{F}(i)}\left(\varvec{A}_{i}-\varvec{A}_{k}\right),$$

5 $$\frac{\partial Err}{\partial {{\varvec{S}}}_{j}}=\sum_{i=1}^{m}I\left(\varvec{X}_{ij}\ne \mathrm{NA}\right)\left(\varvec{X}_{ij}-{{\varvec{A}}}_{i}{{\varvec{S}}}_{j}\right){{\varvec{A}}}_{i}+{\lambda }_{\mathrm{S}}{{\varvec{S}}}_{j}.$$

While single-omics missing value imputation methods have been extensively studied^4,11^, the research of multi-omics integrated missing value imputation strategy is relatively recent. The contributions of the work presented here are three-fold: (1) we elaborate how additional information on the same samples can benefit missing value imputations; (2) we coin the term Fused Regularization to represent the local similarity constraints on imputation functions, and we mathematically illustrate how to design a matrix factorization objective function with fused regularization; and (3) the proposed method is quite general and can be easily extended to incorporate other contextual information such as pathological or clinical scores, etc.

### Introduction to CAM method

**Figure 5:**
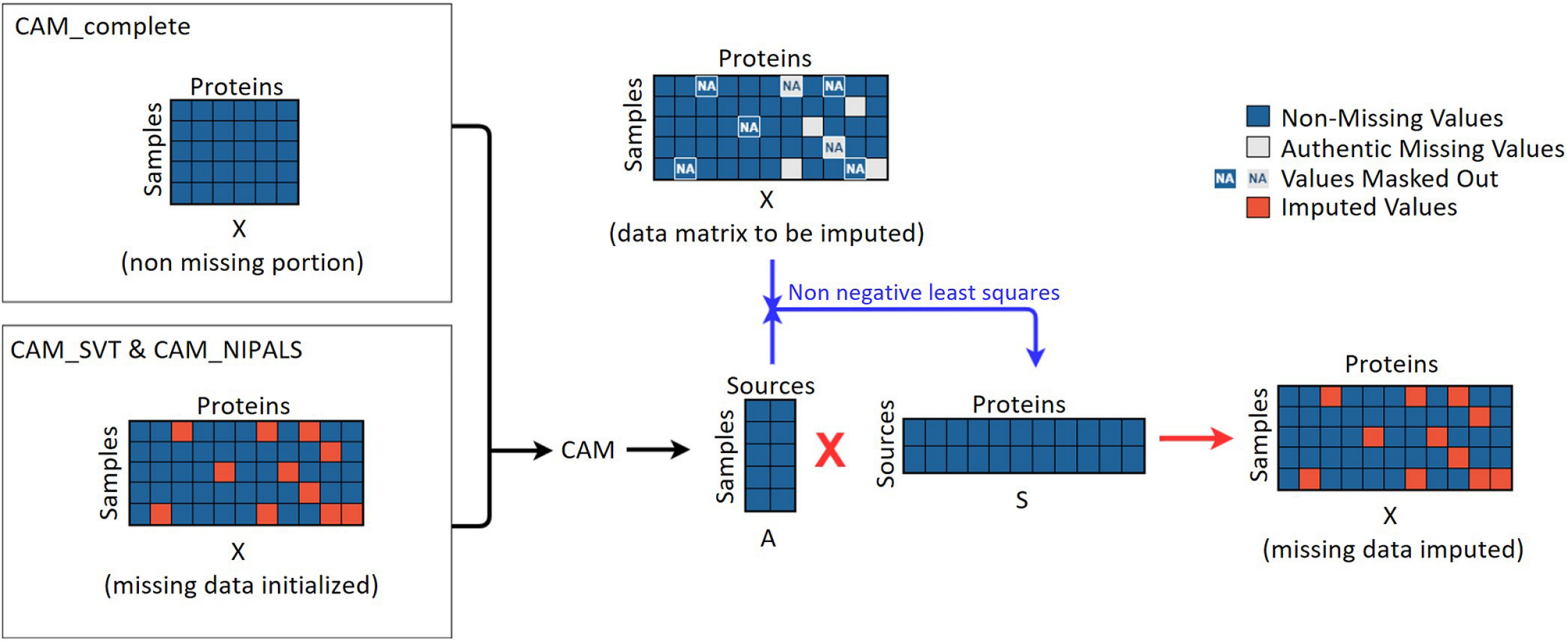
Workflow of the CAM based imputation method with two variant algorithms.

CAM is a latent variable modeling and deconvolution technique previously used for identifying biologically-interpretable cell subtypes or biological archetypes $\varvec{S}_{l\times n}$ and their composition $\varvec{A}_{m\times l}$ in complex tissue ecosystems^6,14,15,21^. We adopted the CAM framework into Eq. 1 and demonstrate that hybrid CAM_SVT and CAM_NIPALS can effectively manage missing values and that this combination leads to a novel and biologically-plausible imputation strategy. The workflow of CAM based method with three variants is given in Fig. 5. Below we present a brief introduction to the CAM principle. Detailed mathematical descriptions and algorithms can be found in our previous publications^14,15^.

The functions of complex tissues are orchestrated by a productive interplay among many specialized cell subtypes or task archetypes^33^. These biological components interact with each other to create a unique physiological or pathophysiological state. To characterize this ground yet dynamic state, mathematical deconvolution of bulk tissue data has been used to model biological tissues as an aggregate of distinct cell or molecular subtypes. The primary objective of mathematical deconvolution is to computationally detect subtype-specific markers, determine the number of constituent subtypes, calculate subtype proportions in individual samples, and estimate subtype-specific expression profiles^34^. Supported by advanced machine learning algorithms and proven theorems, unsupervised deconvolution methods can decompose the mixed molecular signals into many latent variables; these subtypes are biological interpretable and functionally enriched^6,14,35,36^.

**Figure 6:**
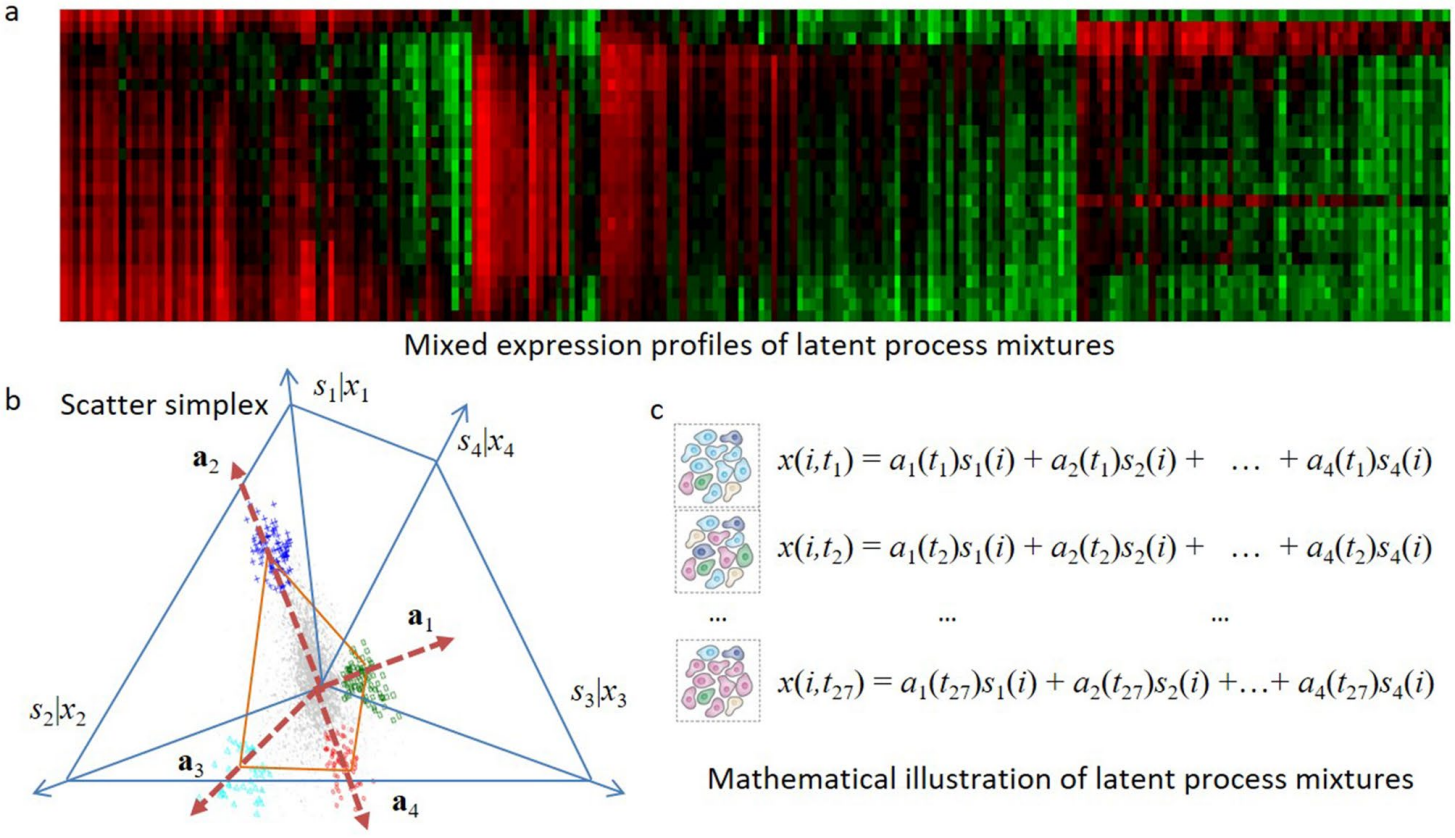
CAM principles for latent variable modelling and deconvolution. (**a**) Mixed expression profile of latent process mixtures. (**b**) Illustration of mixing operation in scatter space, where a compressed and rotated scatter simplex whose vertices host marker genes is produced and corresponded to mixing proportions. (**c**) Mathematical description of expression profile of latent process mixtures.

The CAM pipeline is built on the strong parallelism between nonnegative latent variable models and the theory of convex sets (Fig. 6)^14,37,38^. Tissue samples to be modeled contain an unknown number and varying proportions of molecularly distinctive subtypes (Fig. 6a). Molecular expression in a specific subtype is modeled as being linearly proportional to the abundance of that subtype. We showed that the scatter simplex of bulk data is a rotated and compressed version of the scatter simplex of subtype expressions (Fig. 6b). According to the theory of convex sets^37^, every molecular feature within the scatter simplex can be uniquely determined by the nonnegative combination of the vertices. Thus, the number of the vertices corresponds to the number of molecularly distinctive subtypes present in the bulk samples and the molecular features residing at the vertices are the molecular markers defining such subtypes^14^. CAM works by detecting the vertices of the scatter simplex geometrically, *i.e.*, determining the multifaceted simplex that most tightly encloses the globally measured expression mixtures. Subsequently, the molecular markers residing at the vertices are first identified, and the proportions and specific expression profiles of constituent subtypes are then estimated^14^. The number of latent components is determined by the minimum description length (MDL) criterion, given by

6 $$MDL\left(k\right)=\frac{1}{2}\mathrm{log}\left({\sum }_{j=1}^{n}{\Vert {\varvec{x}}\left(j\right)-\mathbf{A}\mathbf{s}\left(j\right)\Vert }_{2}^{2}\right)+\frac{\left(k-1\right)m}{2}\mathit{log}\left({n}_{MG}\right)+\frac{kn}{2}\mathit{log}\left(m\right),$$

where $k$ is the number of latent components, and ${n}_{MG}$ is the number of marker proteins

.

## Supplementary Information

Supplementary Information.

## Data Availability

The scripts used in the paper is available in R script ProImput. Code for all experiments can be found in the vignette at https://github.com/MinjieSh/ProImput. The operation system can be any system supporting R language.
