# Supplementary material for: Comparative assessment and novel strategy on methods for imputing proteomics data
Source: Sci Rep. 2022 Jan 20;12:1067. doi: 10.1038/s41598-022-04938-0 (PMC8776850; doi:10.1038/s41598-022-04938-0)
Supplement: Supplementary file 1 — Supplementary Information. [file 41598_2022_4938_MOESM1_ESM.docx]

Supplementary Information

Comparative assessment and novel strategy on methods for imputing proteomics data

Minjie Shen, Yi-Tan Chang, Chiung-Ting Wu, Sarah J Parker, Georgia Saylor, Yizhi Wang, Guoqiang Yu, Jennifer E. Van Eyk, Robert Clarke, David M. Herrington, and Yue Wang

**Contents**

[1. Introduction 1](#_Toc65582710)

[2. Experimental Design 2](#_Toc65582711)

[2.1 Real proteomics data 2](#_Toc65582712)

[2.2 Simulation procedure for setting 1 2](#_Toc65582713)

[2.3 Simulation procedure for setting 2 4](#_Toc65582714)

[3. Results 5](#_Toc65582715)

[3.1 Comparative assessment focused on assumed missing mechanisms 5](#_Toc65582716)

[3.2 Comparative assessment focused on authentic missing values 8](#_Toc65582717)

[4. Outlook discussion 9](#_Toc65582718)

[Supplementary Tables 11](#_Toc65582719)

[R Scripts 12](#_Toc65582720)

[References 20](#_Toc65582721)

## Introduction

Existing literature suggests that different imputation methods should be used for different types of assumed missing value mechanisms (Dabke, et al., 2020; Ma, et al., 2020). However, the simulated or biased datasets used in most comparative studies offer few consistent conclusions about the proper use of imputation methods. Many imputation evaluations used model-based or biased (replacing high-intensity data) simulated datasets, with missing values introduced artificially in ways that do not necessarily reflect the missing values in real datasets (Liu and Dongre, 2020). Moreover, the value of integrating multiple data types or biological principles for missing value imputation is largely underappreciated.

Missing values will be a combination of different mechanisms and true missing mechanisms for any given feature are unknown. Acknowledging the limitations of data-driven assessment methodologies, and we developed simulated datasets that reflect the missing value profile of real datasets. Based on our preliminary results obtained using newer imputation strategies, we offer some recommendations on future development directions for improving imputation accuracy.

## Experimental Design

### 2.1 Real proteomics data

Vascular tissue was obtained during autopsy from male and female subjects of any race without any prior diagnosis of cardiovascular disease within 48h of death. In total, up to 1g segments were collected from two standardized regions of the left anterior descending coronary artery (LAD), the abdominal aorta (AA), and one segment of thoracic aorta. A trained pathologist scored each specimen for surface involvement of fatty streak or fibrous plaque/calcified lesions (Parker, et al., 2020). Samples were graded in percentage by pathologists to capture the intimal surface involvement of the atherosclerotic changes. Five tissue subtypes were scored, *i.e.,* fatty streaks (FS), fibrous plaques (FP), complicated lesions (CO), calcified lesions (CA), and normal (NL) (Herrington, et al., 2018).

The TRIC algorithm was used to align all peptide identifications across the full unselected specimen acquisition sets. Fragment level area under the curve data from each file were normalized to the total extracted MS2 signal intensity of that file ((e.g., analogous to normalizing individual signals to total protein in a Western blot). Normalized fragment intensity data were input into the mapDIA software for selection of high-quality fragments and aggregation of fragment level data into peptide intensities, and subsequent peptide level (Parker, et al., 2020).

### 2.2 Simulation procedure for setting 1

Datasets containing no missing values are widely used in discovering the relationship between imputation performance and missing mechanisms, where well-controlled artificial missing values are introduced to the non-missing dataset under various missing mechanism assumptions(Lazar, et al., 2016; Wei, et al., 2018). Since real proteomics data always contain missing values, it is common to filter out proteins with at least one missing value, and use only non-missing proteins in any subsequent imputation analysis. This “non-missing protein” based realistic simulation is used in our first comparison scenario.

A small flexible simulation program based on the R package *imputeLCMD* was written to generate user specified missing rates, quantile cut-off threshold, and mixing proportion of MCAR and MNAR, consistent with the observed relationship between protein missing rate and protein mean intensity in the full LAD 45 DIA data (Lazar, et al., 2016). The *imputeLCMD* has two parameters,

$$\alpha=\frac{n_{\mathrm{MNAR}}+n_{\mathrm{MCAR}}}{n},$$

$$\beta= \frac{n_{\mathrm{MNAR}}}{n_{\mathrm{MNAR}}+n_{\mathrm{MCAR}}}$$

where $n_{\mathrm{MNAR}}$ is the number of MNAR missing values, $n_{\mathrm{MCAR}}$ is the number of MCAR missing values, $\alpha$ is the missing rate, and $\beta$ is the proportion of MNAR. MNAR and MCAR missing values are sequentially introduced into the observed data (Lazar, et al., 2016). Low abundance proteins are censored with a probability of $\beta\alpha$, and the remaining proteins are randomly assigned ‘not available’ with a probability of $(1-\beta)\alpha$. In our study, we selected three of the most representative MNAR proportion ($\beta$) for the non-missing-protein based simulation ($\beta=0, \beta=0.1, \beta=1$), each $\beta$ corresponds to a typical missing mechanism (pure MCAR, Mix-of-MCAR-and-MNAR, and pure MNAR).

The missing pattern(s) present in real proteomics data show that most of the detected (non-missing) signals are from mid/high abundance proteins. Since MNAR/LLOD missing data only appear for low-abundance proteins, the data used in the non-missing-protein based simulation are less likely to have MNAR missing data. For the Mix missing mechanism simulations, we assume that the missing values contain both MCAR missing values and MNAR missing values, where MCAR missing values constitute the majority, and MNAR the minority, of total missing values. This assumption is based on the inspection of the real missing value characteristics in the full data matrix observed showing in Figure S5 (left panel).

Missing values are generated under two assumed missing mechanisms (MCAR and MNAR) following the same procedure proposed in the *imputeLCMD* R package (Lazar, 2015). MCAR missing values are introduced by randomly replacing the data points in the full dataset with missing values. MNAR testing data are introduced by applying a soft quantile cut-off to the full dataset. We generate a normal distribution where the mean is the quantile of the full data, and construct a threshold matrix that has the same size as the original full dataset by randomly drawing values from this normal distribution. We then compared the original full dataset with the threshold matrix and censored the smaller data points in the original full dataset.

The presence of mixed missing patterns can be illustrated by the complex relationship between protein missing rate and protein mean intensity (Webb-Robertson, et al., 2015). Most high-abundant proteins have few missing values when compared with low/mid-range abundant proteins. Furthermore, missing values with varying missing rates arise from a mix of multiple missing mechanisms.

### 2.3 Simulation procedure for setting 2

Authentic missing patterns are lost or ignored if only the non-missing protein values are analyzed. To retain the missing pattern of authentic missing values, we used the full set of real data that contains all proteins (missing rate less than 80%), not only the non-missing proteins but also authentic missing proteins. In our second series of simulations, we added a small number of masked missing values (a small perturbation) to the authentic missing values. The imputation performance is evaluated based on these set-aside masked testing data. While it is difficult to completely capture the true missing pattern(s) in the real dataset, this “all protein” based simulation is a good complement to the traditional approach (non-missing-protein-only based analyses).

Because either pure MCAR or pure MNAR testing data can distort the global missing pattern, to preserve the global missing pattern as much as possible, we propose a new method to introduce artificial missing values called Mimicking-real-pattern. The testing data are introduced protein-wise, for each protein, the number of introduced missing values is based on the original protein missing rate. If the original protein has no missing data, it will remain non-missing; if the original protein has large missing rate, more artificial missing data will be introduced to it proportionally. This is achieved by multiplying a coefficient to the original protein missing rate to obtain the masked protein missing rate, and masking the full data set according to these ‘proportional’ masked protein missing rates.

Combination of MNAR and Mimicking-real-pattern likely have a large impact on the local missing pattern (protein missing rate) since a large number of empty proteins may be produced under these two mechanisms (MNAR and adaptive-rate masked value). To prevent the negative impact of empty proteins, we keep several data points in each empty protein appeared after introducing the artificial missing data. The values of the remaining data points would make a difference to the NRMSE trend across different missing rates, so we keep a few of the largest values for the MNAR mechanism and a few random values for other mechanisms. The characteristic relationship of the resulting total missing rate (authentic and masked) versus mean expression is shown in Figure S5 (right panel), clearly preserving well the overall patterns of the original full data matrix.

## Results

### 3.1 Comparative assessment focused on assumed missing mechanisms

As shown in **Figure S1**, the performance pattern under Mix assumption is similar to that of the MCAR assumption in the non-missing-protein scenario because the majority of missing data are MCAR missing data. Among all the selected methods, NIPALS and SVT performed the best for all criteria (NRMSE, RMSE, SOR), while the MNAR-devoted method HalfMin performed the worst, as expected. Compared with the MCAR performance pattern, the presence of MNAR missing data affects the performance trend across total missing rates, such that the RMSE goes down when the total missing value rate increases.


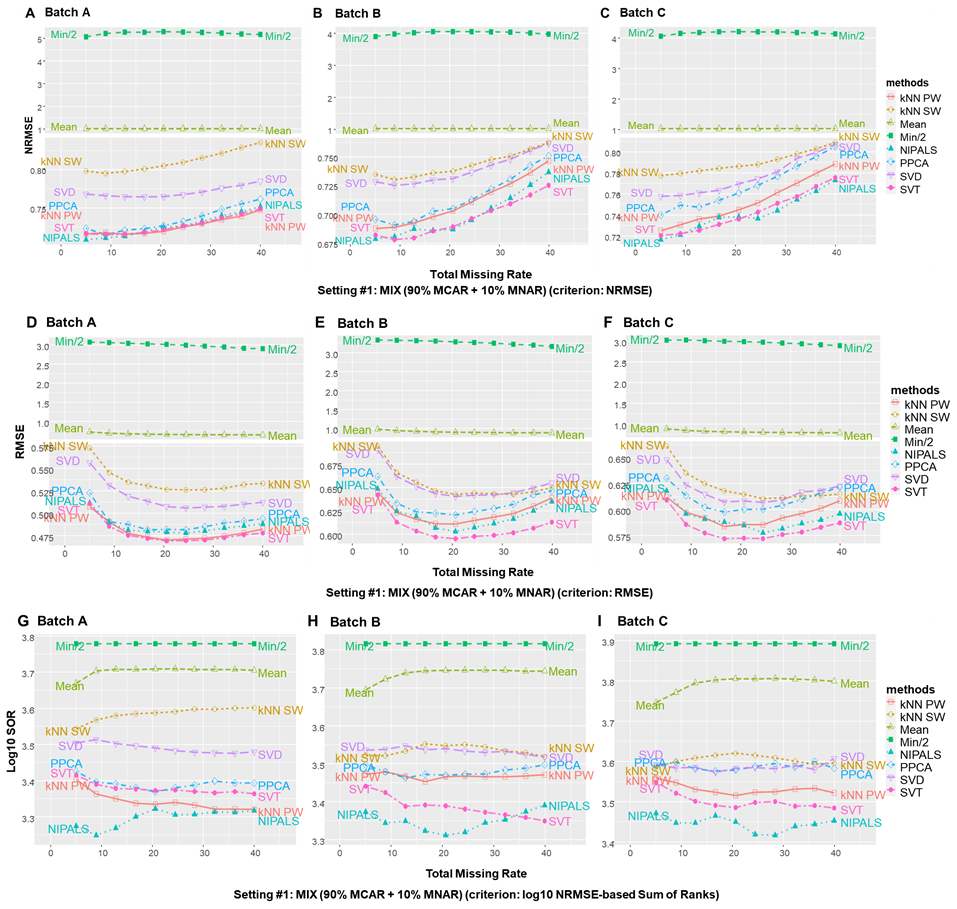


**Figure S1.** The performance trends under MIX assumption across three different batches of experimentally acquired proteomics data.

As shown in **Figure S2**, the performance trends under MNAR assumption are similar across different batches of experimentally acquired proteomics data. More details are reported in the main body of the paper.


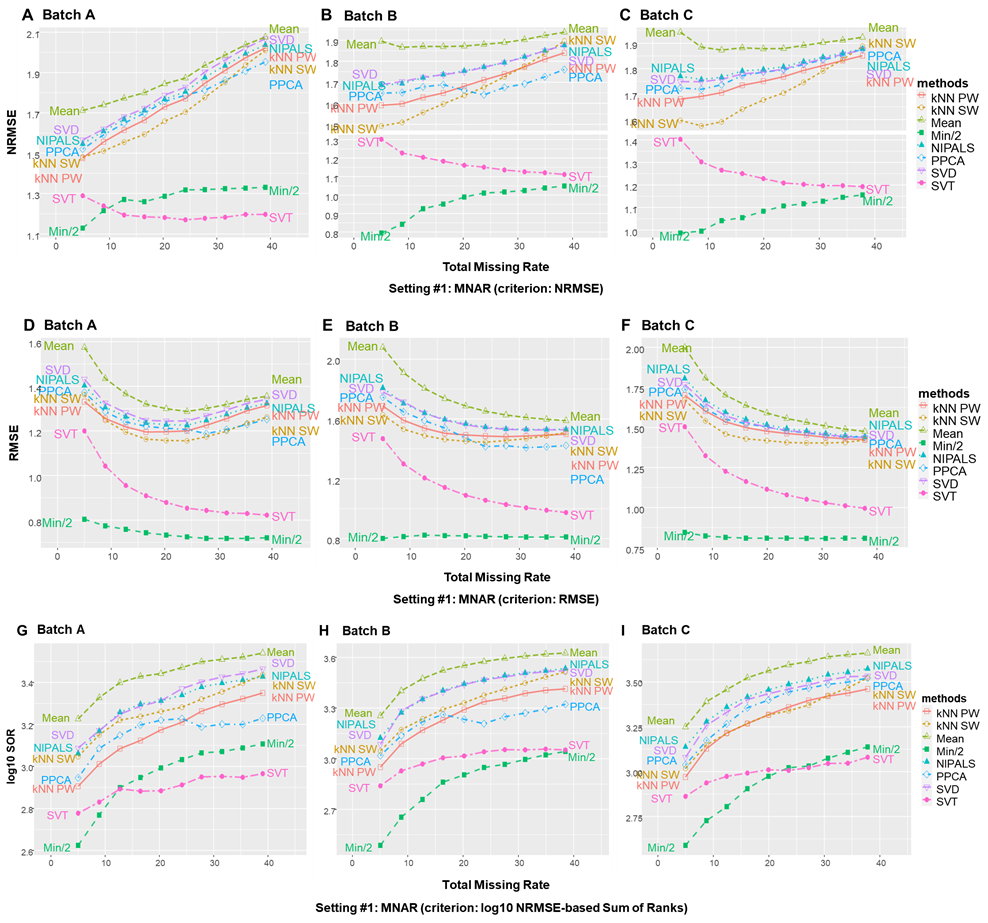


**Figure S2.** The performance trends under MNAR assumption across three different batches of experimentally acquired proteomics data.


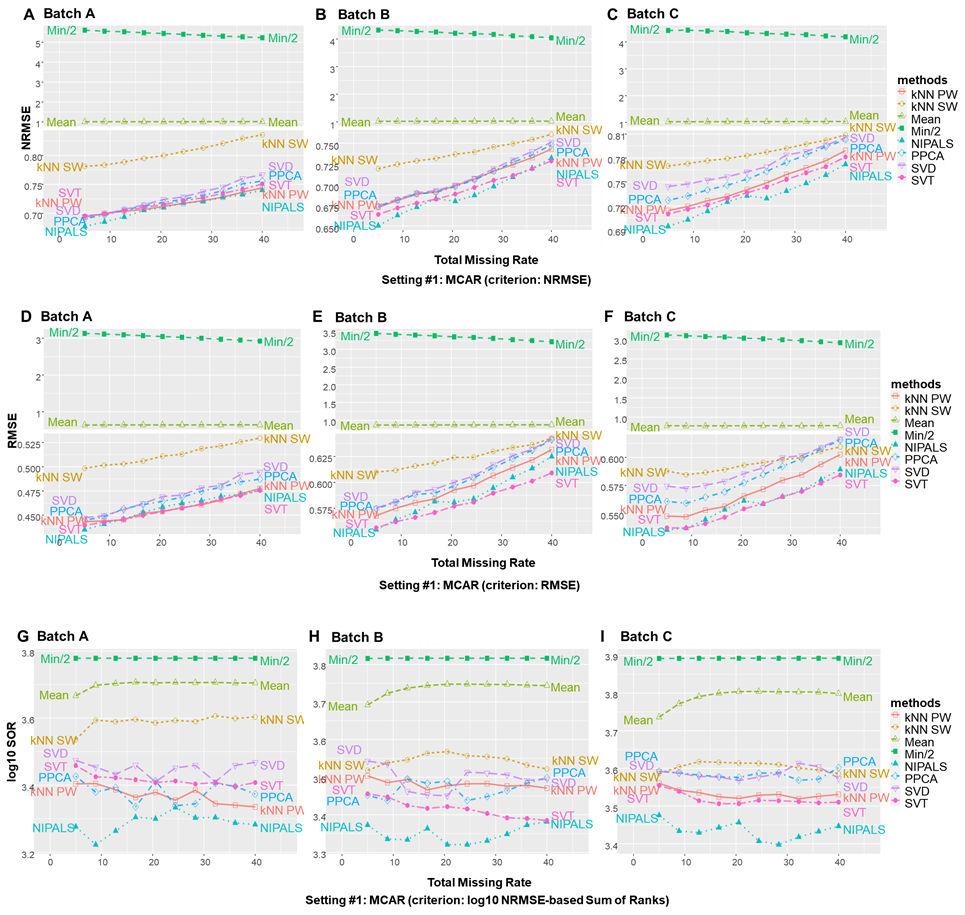


**Figure S3.** The performance trends under MCAR assumption across three different batches of experimentally acquired proteomics data.

### 3.2 Comparative assessment focused on authentic missing values

As shown in **Figure S4**, the performance trends under simulation setting #2 are similar across three different batches of experimentally acquired proteomics data.


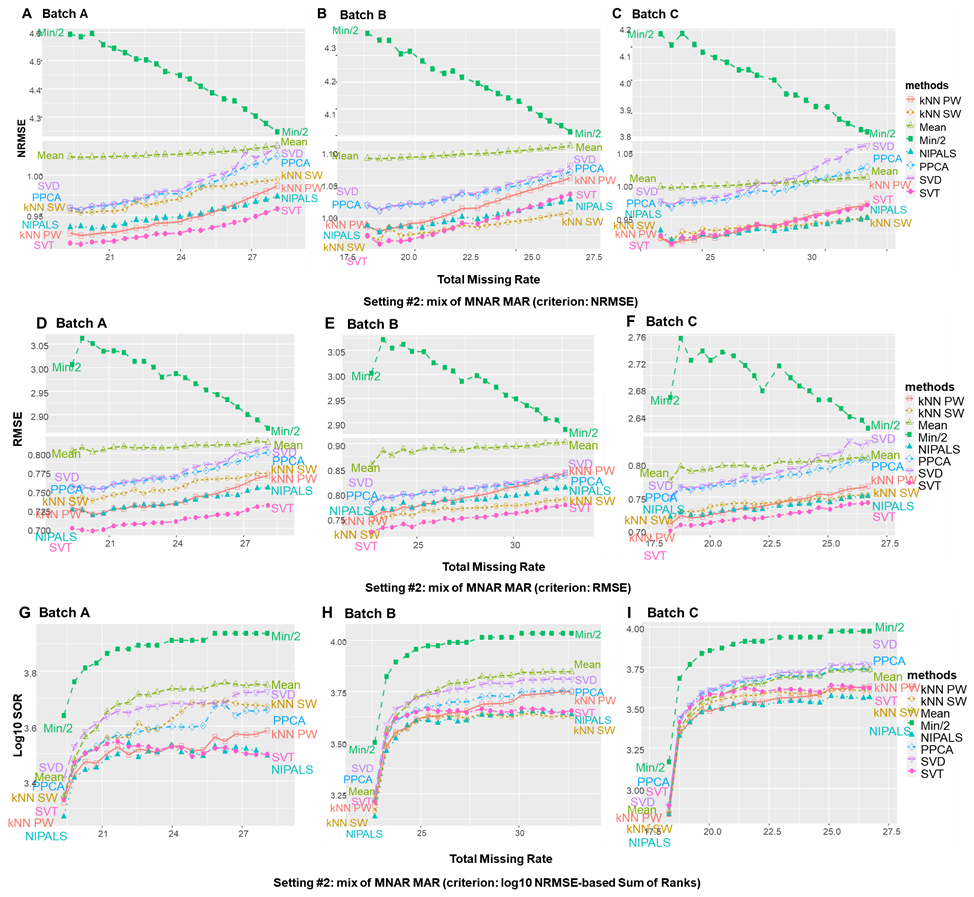


**Figure S4.** The performance trends under simulation setting #2 across three different batches of experimentally acquired proteomics data.

### 3.3 Additional experimental results


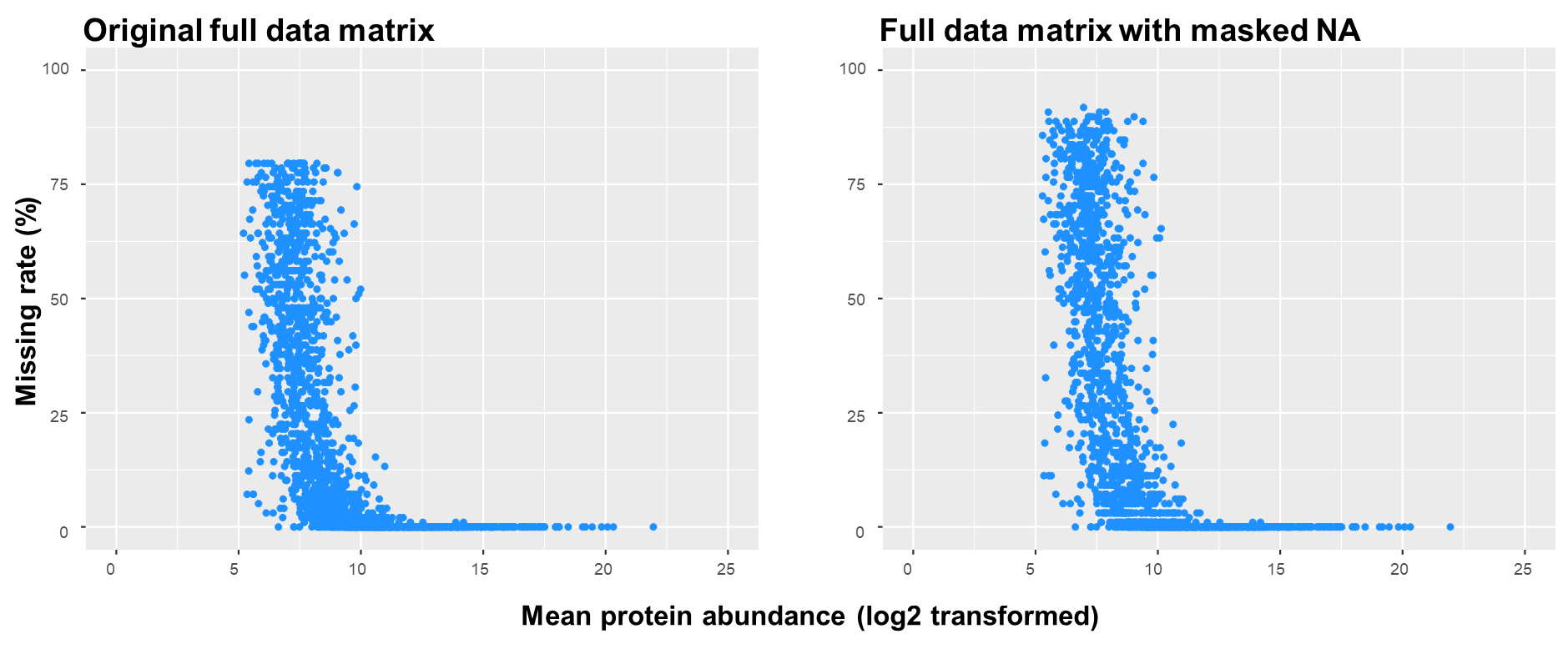


**Figure S5.** The overall pattern of missing values illustrated by the relationship between protein missing rate and protein mean intensity, before (left panel) and after (right panel) introducing masked NA.


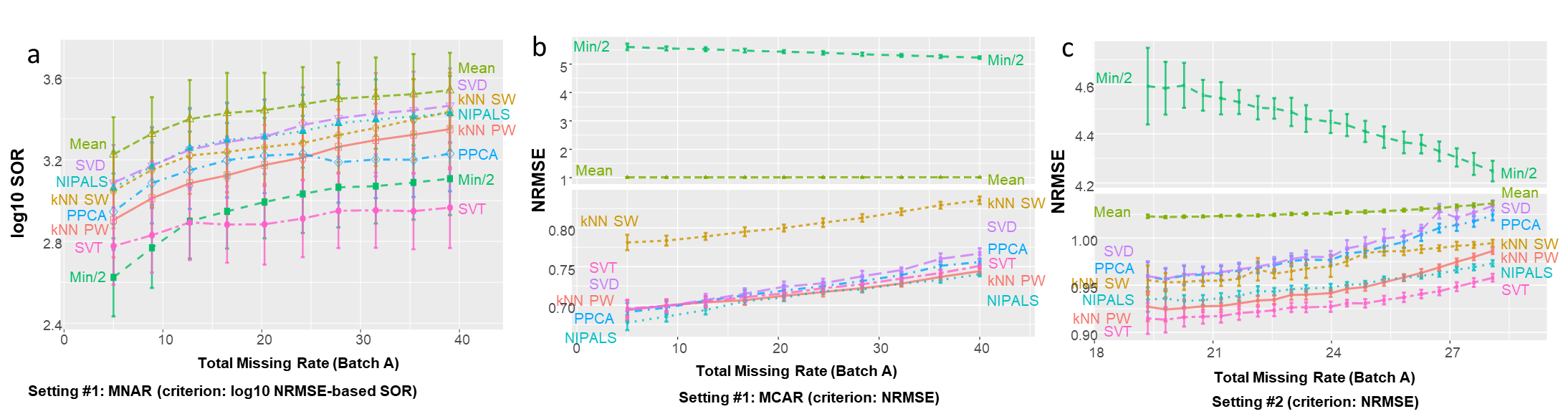


**Figure S6.** **Imputation performance with error bars** (standard deviation of the sampling distribution over 50 trials)**.** (a) Imputation performance of the eight methods on the simulation data of setting #1, with assumed MNAR missing mechanism and varying total missing rates. (b) Imputation performance of the eight methods on the simulation data of setting #1, with assumed MCAR missing mechanism and varying total missing rates. (c) Imputation performance of the eight methods on the simulation data of setting #2, with varying total missing rates.


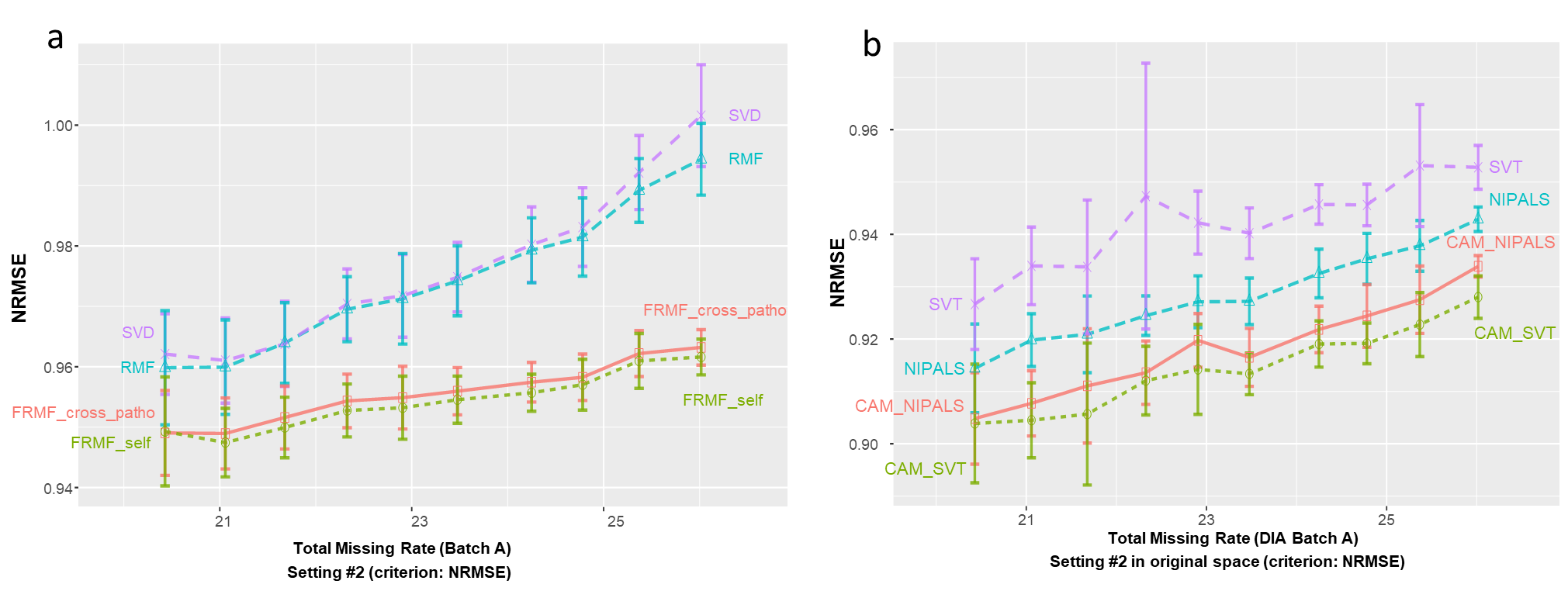


**Figure S7.** **Imputation performance with error bars** (standard deviation of the sampling distribution)**.** (a) Imputation performance of the FRMF variants on the simulation data of setting #2, with varying masked rates, over 30 trials. (b) Imputation performance of the CAM variants on the simulation data of setting #2 in original space, with varying masked rates, over 10 trials.


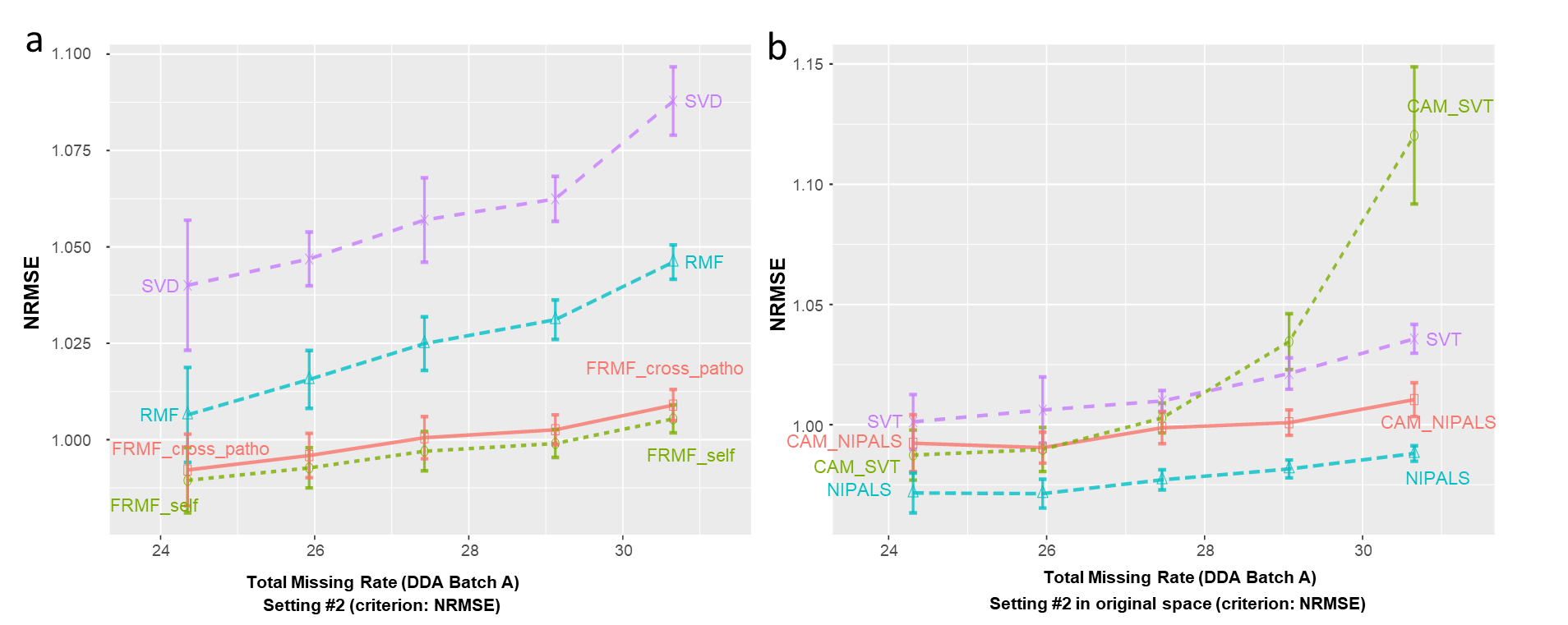


**Figure S8.** **Imputation performance of FRMF and CAM on DDA-simulated dataset with error bars** (standard deviation of the sampling distribution over 10 trials)**.** (a) Imputation performance of the FRMF variants on the simulation data of setting #2, with varying masked rates. (b) Imputation performance of CAM variants on the simulation data of setting #2, with varying masked rates, in comparison to that of Mean, SVT, and NIPALS. The imputation accuracy is evaluated in the original intensity space (before log-transformation).


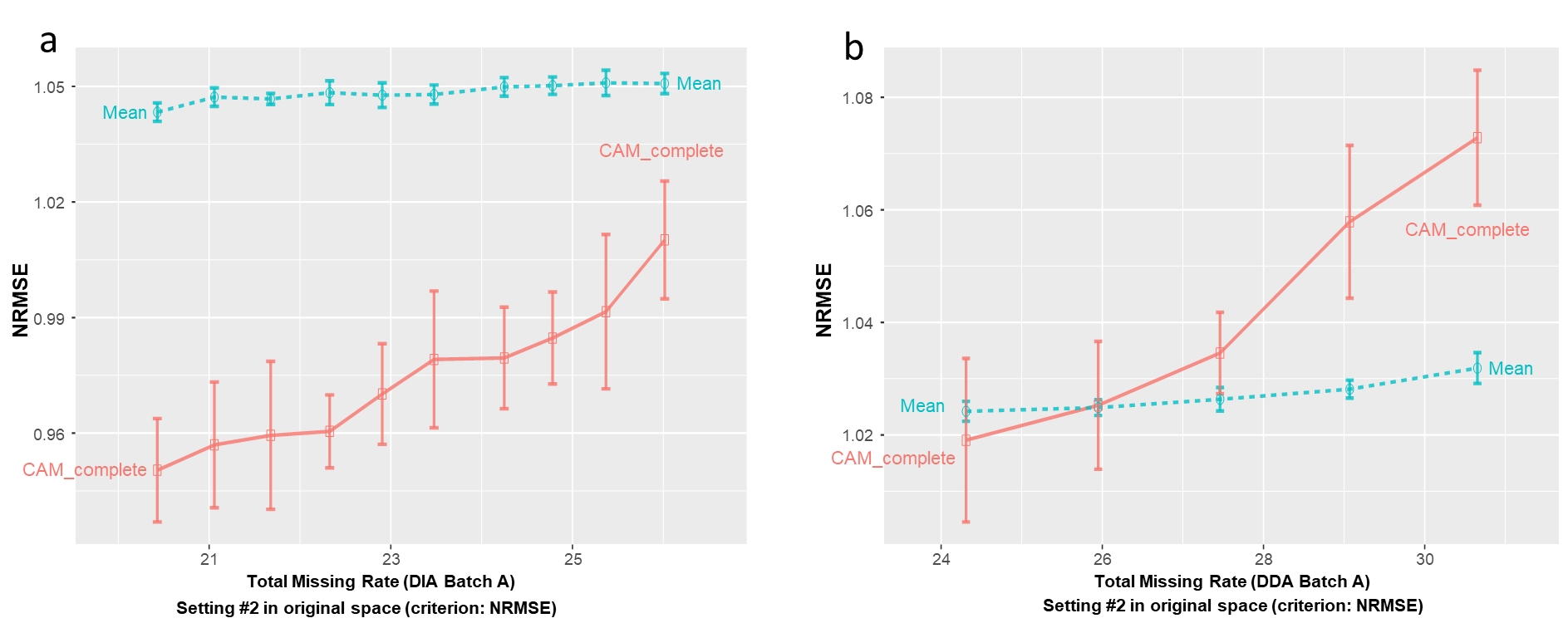


**Figure S9.** **Imputation performance of CAM on DIA/DDA-simulated dataset with error bars** (standard deviation of the sampling distribution over 10 trials)**.** (a) Imputation performance of the CAM without pre-imputation on DIA type, with varying masked rates, (b) Imputation performance of the CAM without pre-imputation on DDA type, with varying masked rates; in comparison to that of Mean method. The imputation accuracy is evaluated in the original intensity space (before log-transformation).

## Outlook discussion

Similar to the CAM concept, Nonnegative Matrix Factorization (NMF) is a technique widely used in blind signal decomposition. Because NMF can handle missing values naturally as SVD/SVT while working in the original intensity space, this property has recently been exploited to determine the rank hyperparameter in low-rank matrix factorization. Thus, it would be an interesting effort to develop a CAM_NMF imputation method, where cross-validation with random entry masking can be used to select the proper rank (Lin and Boutros, 2020).

The set-aside masking is a simple yet effective technique for many applications. Here we only demonstrate its application to evaluate imputation accuracy without altering the characteristics of observed and missing values.

**Supplementary Tables**

**Table S1.** Summary of real proteomics datasets used in this work (DDA-MS) (Herrington, et al., 2018; Parker, et al., 2020).


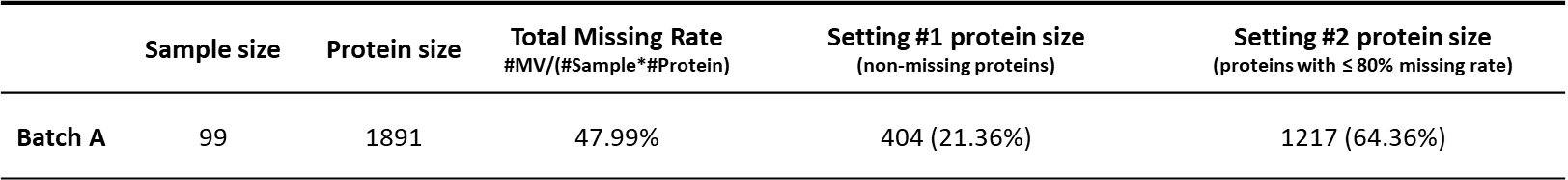


## R Scripts

The scripts below show how to obtain some of the results reported here with certain parameters. More suggests on parameter setting can be found in the package vignette.

**A toy example for CAM Imputation (Setting #2)**

## Set up your working directory

*#setwd("YOUR_PATH/ProImput")*

## Install packages

**source**("Dependencies.R")

## Data

raw_data <- **readRDS**("Data/raw_data_large.rds")

## Data preprocessing

orig_data <- raw_data
smp <- **nrow**(orig_data)
prt <- **ncol**(orig_data)
smp

## [1] 98

prt

## [1] 1935

## Methods involved & Missing rate range

method_name <- **c**("pwMean", "CAM_SVT", "SVT")
display_method_name <- **c**("Mean", "CAM_SVT", "SVT")
number_of_method <- **length**(method_name)
misg_rate <- **seq**(0.15, 0.75, length.out = 5)
number_of_misg_rate <- **length**(misg_rate)

## DO NOT RUN below chunk in the toy example.

*# method_name <- c("pwMean", "CAM_SVT", "SVT", "CAM_NIPALS", "NIPALS", "CAM_cmplt")*
*# display_method_name <- c("Mean", "CAM_SVT", "SVT", "CAM_NIPALS", "NIPALS", "CAM_cmplt")*
*# number_of_method <- length(method_name)*
*# misg_rate <- seq(0.15, 0.75, length.out = 10)*
*# number_of_misg_rate <- length(misg_rate)*

## Load the best parameters

best_parameter <- **matrix**(NA,
 nrow = number_of_misg_rate,
 ncol = number_of_method)
best_parameter[, 3] <- 40000

best_parameter[, **c**(2)] <- 10 *### for toy example*
*# best_parameter[, c(2, 4, 5, 6)] <- 10* ### *for more*

## Number of iterations

iteration <- 1

## Generate the real-data-based simulated missing data

missing_matrix <- **array**(0,
 **c**(
 number_of_misg_rate,
 iteration,
 **nrow**(orig_data),
 **ncol**(orig_data)
 ))

start_time <- **Sys.time**()
**for** (rate **in** 1**:**number_of_misg_rate) {
 **for** (loop **in** 1**:**iteration) {
 misg_data <- **data_with_MV_mask_out**(
 orig_data,
 option = "MAR",
 parameter = misg_rate[rate],
 keep = "max"
 )

 missing_matrix[rate, loop, , ] <- misg_data**$**data_extra_MV
 }
}

end_time <- **Sys.time**()
end_time **-** start_time

## Time difference of 0.661109 secs

total_misg_rate <- misg_rate
**for** (rate **in** 1**:**number_of_misg_rate) {
 total_misg_rate[rate] <-
 100 ***** **sum**(**is.na**(missing_matrix[rate, 1, , ])) **/**
 (**ncol**(orig_data) ***** **nrow**(orig_data))
}

## Imputation (~ 20 minutes for each iteration)

temp <- **list**()
predict_matrix <- **array**(0,
 **c**(
 number_of_misg_rate,
 iteration,
 number_of_method,
 **nrow**(orig_data),
 **ncol**(orig_data)
 ))
start_time <- **Sys.time**()

**for** (i **in** 1**:**number_of_method) {
 **for** (rate **in** 1**:**number_of_misg_rate) {
 **for** (loop **in** 1**:**iteration) {
 skip_to_next <- FALSE

 **tryCatch**({
 temp <-
 **impute**(
 log_data = **log2**(missing_matrix[rate, loop, ,]),
 orig_data = missing_matrix[rate, loop, ,],
 methods = method_name[i],
 parameter = best_parameter[rate, i],
 dim.rdc = 10,
 cluster.num = 35
 )

 **if** (i **%in%** **c**(1, 3)) { *### for toy example*
 *# if (i %in% c(1, 3, 5)) {* ### *for more*
 predict_matrix[rate, loop, i, ,] <-
 2 **^** temp**$**imp
 } **else** {
 predict_matrix[rate, loop, i, ,] <-
 temp**$**imp
 }


 }, error = **function**(error_condition) {
 **message**('Caught an error!')
 **message**(error_condition)
 skip_to_next <- TRUE
 }, finally = {
 **message**('Success!')
 })

 **if** (skip_to_next) {
 **next**
 }
 }
 }
}

end_time <- **Sys.time**()
end_time **-** start_time

## Time difference of 39.3048 mins

## Evaluate using NRMSE

nrmse_matrix <- **matrix**(0,
 nrow = number_of_misg_rate ***** number_of_method,
 ncol = iteration)


start_time <- **Sys.time**()
**for** (rate **in** 1**:**number_of_misg_rate) {
 **for** (loop **in** 1**:**iteration) {
 data_extra_MV <- missing_matrix[rate, loop, ,]
 **for** (i **in** 1**:**number_of_method) {
 data_imputed <- predict_matrix[rate, loop, i, ,]
 nrmse_matrix[(rate **-** 1) ***** number_of_method **+** i, loop] <-
 **data_with_MV_NRMSE**(orig_data, data_extra_MV, data_imputed)
 }
 }
}
end_time <- **Sys.time**()
end_time **-** start_time

## Time difference of 0.633805 secs

## Plot the results

backup <- nrmse_matrix
nrmse_result <- **plot_nrmse**(backup,
 display_method_name,
 total_misg_rate,
 "NRMSE",
 "CAM Workflow (Example)",
 panel = 2)
**print**(nrmse_result)

## Save the plot

**emf**(file = "CAM Workflow (Example).emf",
 width = 8,
 height = 4.8)
**print**(nrmse_result)
**dev.off**()

**A toy example for FRMF Imputation (Setting #2)**

## Set up your working directory

*#setwd("YOUR_PATH/ProImput")*

## Install packages

**source**("Dependencies.R")

## Data

raw_data <- **readRDS**("Data/raw_data_small.rds")

## Data preprocessing

log_data <- **log2**(raw_data)
smp <- **nrow**(log_data)
prt <- **ncol**(log_data)
smp

## [1] 47

prt

## [1] 2325

## Methods involved & Missing rate range

method_name <- **c**("pwMean", "FRMF", "FRMF")
display_method_name <- **c**("Mean", "RMF", "FRMF_self")
number_of_method <- **length**(method_name)
misg_rate <- **seq**(0.15, 0.55, length.out = 5)
number_of_misg_rate <- **length**(misg_rate)

## DO NOT RUN below chunk in the toy example.

*# method_name <- c("pwMean", "FRMF", "FRMF", "FRMF")*
*# display_method_name <- c("Mean", "RMF", "FRMF_self", "FRMF_cross_patho")*
*# number_of_method <- length(method_name)*
*# misg_rate <- seq(0.15, 0.75, length.out = 10)*
*# number_of_misg_rate <- length(misg_rate)*
*# external_info <- readRDS("Data/external_data_small.rds")*

## Load the best parameters

best_parameter <- **matrix**(NA,
 nrow = number_of_misg_rate,
 ncol = number_of_method)

best_parameter[, **c**(2, 3)] <- 3 *### for toy example*
*# best_parameter[, c(2, 3, 4)] <- 3* ### *for more*

## Number of iterations

iteration <- 1

## Generate the real-data-based simulated missing data

missing_matrix <- **array**(0,
 **c**(
 number_of_misg_rate,
 iteration,
 **nrow**(log_data),
 **ncol**(log_data)
 ))

start_time <- **Sys.time**()
**for** (rate **in** 1**:**number_of_misg_rate) {
 **for** (loop **in** 1**:**iteration) {
 misg_data <- **data_with_MV_mask_out**(log_data,
 "MAR",
 misg_rate[rate],
 "max")**$**data_extra_MV

 missing_matrix[rate, loop, , ] <- misg_data
 }
}

end_time <- **Sys.time**()
end_time **-** start_time

## Time difference of 0.5899248 secs

total_misg_rate <- misg_rate
**for** (rate **in** 1**:**number_of_misg_rate) {
 total_misg_rate[rate] <-
 100 ***** **sum**(**is.na**(missing_matrix[rate, 1, , ])) **/**
 (**ncol**(log_data) ***** **nrow**(log_data))
}

## Imputation (~ 35 minutes for each iteration)

temp <- **list**()
predict_matrix <- **array**(0,
 **c**(
 number_of_misg_rate,
 iteration,
 number_of_method,
 **nrow**(log_data),
 **ncol**(log_data)
 ))
start_time <- **Sys.time**()

**for** (i **in** 1**:**number_of_method) {
 **for** (rate **in** 1**:**number_of_misg_rate) {
 **for** (loop **in** 1**:**iteration) {
 **if** (i **==** 3) {
 nbr_info_mat <- missing_matrix[rate, loop, , ]
 } **else** **if**(i **==** 4){
 nbr_info_mat <- external_info
 } **else**{
 nbr_info_mat <- NULL
 }

 skip_to_next <- FALSE

 **tryCatch**({
 temp <-
 **impute**(
 log_data = missing_matrix[rate, loop, , ],
 methods = method_name[i],
 parameter = best_parameter[rate, i],
 nbr_info_mat = nbr_info_mat
 )

 predict_matrix[rate, loop, i, , ] <-
 temp**$**imp


 }, error = **function**(error_condition) {
 **message**('Caught an error!')
 **message**(error_condition)
 skip_to_next <- TRUE
 }, finally = {
 **message**('Success!')
 })

 **if** (skip_to_next) {
 **next**
 }
 }
 }
}

end_time <- **Sys.time**()
end_time **-** start_time

## Time difference of 37.77723 mins

## Evaluate using NRMSE

nrmse_matrix <- **matrix**(0,
 nrow = number_of_misg_rate ***** number_of_method,
 ncol = iteration)


start_time <- **Sys.time**()
**for** (rate **in** 1**:**number_of_misg_rate) {
 **for** (loop **in** 1**:**iteration) {
 data_extra_MV <- missing_matrix[rate, loop, ,]
 **for** (i **in** 1**:**number_of_method) {
 data_imputed <- predict_matrix[rate, loop, i, ,]
 nrmse_matrix[(rate **-** 1) ***** number_of_method **+** i, loop] <-
 **data_with_MV_NRMSE**(log_data, data_extra_MV, data_imputed)
 }
 }
}
end_time <- **Sys.time**()
end_time **-** start_time

## Time difference of 0.4231861 secs

## Plot the results

backup <- nrmse_matrix
nrmse_result <- **plot_nrmse**(backup,
 display_method_name,
 total_misg_rate,
 "NRMSE",
 "FRMF Workflow (Example)",
 panel = 5)
**print**(nrmse_result)

## Save the plot

**emf**(file = "FRMF Workflow (Example).emf",
 width = 8,
 height = 4.8)
**print**(nrmse_result)
**dev.off**()

## References

Dabke, K.*, et al.* A Simple Optimization Workflow to Enable Precise and Accurate Imputation of Missing Values in Proteomic Datasets. *bioRxiv* 2020.

Herrington, D.M.*, et al.* Proteomic Architecture of Human Coronary and Aortic Atherosclerosis. *Circulation* 2018;137(25):2741-2756.

Lazar, C. imputeLCMD: A collection of methods for left-censored missing data imputation. In.; 2015.

Lazar, C.*, et al.* Accounting for the multiple natures of missing values in label-free quantitative proteomics data sets to compare imputation strategies. *Journal of proteome research* 2016;15(4):1116-1125.

Lin, X. and Boutros, P.C. Optimization and expansion of non-negative matrix factorization. *BMC Bioinformatics* 2020;21(1):7.

Liu, M. and Dongre, A. Proper imputation of missing values in proteomics datasets for differential expression analysis. *Brief Bioinform* 2020.

Ma, W., al., e. and Wang, P. DreamAI: algorithm for the imputation of proteomics data. *bioRxiv* 2020.

Parker, S.J.*, et al.* Identification of Putative Early Atherosclerosis Biomarkers by Unsupervised Deconvolution of Heterogeneous Vascular Proteomes. *J Proteome Res* 2020;19(7):2794-2806.

Webb-Robertson, B.-J.M.*, et al.* Review, evaluation, and discussion of the challenges of missing value imputation for mass spectrometry-based label-free global proteomics. *Journal of proteome research* 2015;14(5):1993-2001.

Wei, R.*, et al.* Missing value imputation approach for mass spectrometry-based metabolomics data. *Scientific reports* 2018;8(1):1-10.
